# What style of leadership do women in STEMM fields perform? Findings from an international survey

**DOI:** 10.1371/journal.pone.0185727

**Published:** 2017-10-05

**Authors:** Meredith Nash, Amanda Davies, Robyn Moore

**Affiliations:** 1 School of Social Sciences, University of Tasmania, Hobart, Tasmania, Australia; 2 School of Built Environment, Curtin University, Perth, Western Australia, Australia; Ruhr-Universitat Bochum, GERMANY

## Abstract

It is widely acknowledged that women in science, technology, engineering, mathematics, and medicine (STEMM) fields are underrepresented in leadership globally. However, little is known about how leadership styles of women in STEMM relate to this underrepresentation. This article discusses findings from a survey examining how 61 women in STEMM define leadership and describe their own leadership styles. Using content analysis and drawing on Full Range Leadership Model factors, findings suggest that women define leadership and describe their own leadership styles using transformational factors. However, there was no consistency in how participants defined ideal leadership or how they defined their own leadership styles. This finding unsettles ideas of distinctly gendered leadership styles. We argue that expectations that leadership will be performed in distinctly gendered styles may be contributing to the underrepresentation of women in leadership roles in STEMM.

## Introduction

If those who make selection and promotion decisions believe that women's leadership styles are different from men's …or that women should not manifest certain particularly effective leadership styles …the path to leadership may become more difficult for women than men [1].

Leadership scholars and commentators have argued there is a difference between the ‘typical’ leadership styles of men and women [1,2]. This truism has been in circulation for over 25 years, arguably, since the publication of Rosener’s ‘Ways Women Lead’ in the *Harvard Business Review* [3]. Some commentators further argue that this difference in leadership style makes one gender more suitable than the other for particular leadership roles. For instance, women are considered to be more suitable in scenarios demanding team work, whereas men are perceived to be more effective in situations requiring authority [4,5]. This article focuses on women in science, technology, engineering, mathematics, and medicine (STEMM) fields. Globally, within STEMM fields, women continue to be underrepresented in leadership positions, particularly in high-level or elite leadership roles.

The underrepresentation of women leaders in STEMM fields has not gone unnoticed. For more than two decades, a range of programs have been developed to provide additional support to women in STEMM to move through the ‘pipeline’ (for example, ADVANCE in the US; Athena SWAN in the UK and Australia). STEMM specific programs recognise the impact of organisational context on leadership opportunities and pathways. However, little attention has been given to recording and critically assessing the leadership styles of women in STEMM and how these might relate to the continued underrepresentation of women in STEMM leadership. This article adds critical knowledge to the wider discussions concerning gender imbalances in STEMM leadership. We investigate a key question: Is there a typical style of leadership exercised by women in STEMM?

This paper reports the results of a survey of 61 women in STEMM fields and seeks to identify how they define leadership and describe their own leadership styles. We argue that expectations that leadership will be performed in distinctly gendered styles may be contributing to the underrepresentation of women in leadership roles in STEMM.

## Literature review

### Gendered differences in leadership styles

Many studies have been published that examine the role gender might have on the nature and effectiveness of leadership [6]. Studies that account for contextual variables such as age, education, and occupation lend support to the position that there is a gender difference [7–9]. Studies that focus on the adoption of different leadership styles have also found gender differences [1,10]. Studies reporting a gender difference in leadership styles often ascribe collaborative and participatory styles to women while men are most often reported as having or using direct and controlling styles [11–13]. However, as Powell concludes from a summary of meta-analyses, there are limitations in studies examining gendered differences in leadership styles: fewer gendered differences in leadership style are found in studies of actual leaders than laboratory studies or studies of non-leaders who are asked how they would behave if they were leaders [14].

Overall, the existing leadership literature reinscribes ideas of essential differences between men and women in terms of leadership styles. For example, in their discussion of barriers for women in exercising leadership, Sanchez-Hucles and Davis argue that male leadership is too often characterised as ‘command and control’, whereas female leadership is portrayed as ‘facilitative and collaborative’ [15]. In some circumstances, perceiving leadership styles through a gendered lens can benefit women. For example, female leadership may be considered an asset in the current climate with flatter organisational structures, team-based management, and increased globalisation [16–18]. However, as suggested by the quote at the beginning of this paper, persistent belief in gendered leadership styles often impedes women’s access to leadership roles. For example, Marinelli and Lord note that interpersonal and relational aspects of leadership, in which women are perceived as excelling, are not reflected in existing leadership frameworks [19]. Consequently, narrow readings of women’s leadership styles impair women’s capacities to lead [15].

One of the factors underpinning the lack of clear agreement within the leadership scholarship about the relationship between gender and leadership styles has been that ‘leadership has traditionally been studied using [putatively] masculine norms as the standards for behaviors’ [7]. To address this, scholars have examined the relationship between gender and leadership using Bass and Avolio’s Full Range Leadership Model (FRLM) which incorporates leadership styles that are considered typical of either gender. The FRLM encompasses transformational, transactional, and non-transaction (laissez-faire) styles and is regarded as a leading model for describing leadership styles [20]. The model was first proposed by Avolio and Bass [21] and expanded on earlier work by Burns [22] who identified a transformational style of leadership. The FRLM model originally proposed by Bass and Avolio contained five factors [23]. However, as the evidence base regarding leadership traits expanded, they revised their model to include nine factors [24]. Five of the nine factors describe transformational leadership, three describe transactional leadership, and one describes laissez-faire leadership, as shown in Table 1. Our descriptions are based on Barbuto *et al*., Antonakis *et al*., and Lauber [7, 24, 25].

**Table 1:** Full Range Leadership Model and factor descriptions used for the analysis.

Leadership Type	Leadership Factor	Description
Transformational	Inspirational motivation	motivate those around them by providing a vision and meaning for the work undertaken by followers. display optimism and generate enthusiasm and individual/ team spirit has clear goals and positive attitude for future
	Idealized influence as an attribute	demonstrates attributes that motivate respect and pride by association charisma focuses on on higher-order ideals and values. Followers build close emotional ties to the leader. Leader is trustworthy and inspires confidence in followers
	Idealized influence as a behavior	communicates values, purpose and importance of mission emphasises collective sense of mission and values, and acts upon these values.
	Intellectual stimulation	examines new perspectives on problem solving and task completion challenges assumptions of followers`beliefs, their analysis of problems faced, face and solutions generated.
	Individualised consideration	act as coach/mentor by paying attention to individual needs for achievement and growth. focuses on developing followers’ skills recognizes individual aspirations
Transactional	Contingent reward	clarify expectations for followers recognises/rewards followers when goals are achieved.
	Active management-by-exception	actively attends to deviations from rules to avoid these deviations; if necessary, corrective actions are taken
	Passive management-by-exception	waits until problems are severe before intervening intervenes only after errors are detected or standards are not met
Non-transactional	Laissez-faire	exhibits widespread absence and lack of involvement during critical junctures

Crucially, the factor or combination of factors required for a leader to be effective is dependent on the context in which the leader is performing. A meta-analysis by Lowe *et al*. concludes that there are multiple combinations of factors that relate to effective leadership and these combinations are moderated by contextual factors including organisation type and leadership role [26]. To our knowledge, no published studies identify relevant factors for leadership in STEMM organisations, despite considerable investment in leadership training and development.

In line with the finding that context influences leadership style, researchers have examined the role of gender in influencing leadership style using the FRLM. A meta-analysis of leadership studies by Eagly *et al*. reveals that effective female leaders tended to perform factors associated with transformational leadership more than male leaders [16]. Barbuto *et al*. used the FRLM to show that gender only affected leaders’ use of transformational factors if leaders’ education had not progressed beyond high school. These differences diminished as leaders’ education levels increased. The combination of age and gender did not produce an overall main effect. They conclude that ‘if the contextual nature of gender differences had not been a focus of the present study, we would have concluded inaccurately that no gender difference existed’ and suggest that earlier studies examining the relationship between gender and leadership be re-evaluated to consider the contextual nature of gender differences [7]. Antonakis et al. observe that leaders adjust their behavior depending on the environment in which leadership is exercised. However, in homogenous environments, Antonakis *et al*. found that leaders enact different behaviours depending on gender, with women exhibiting higher levels of individualised consideration and men adopting higher levels of management by exception [24]. This is an interesting result as it appears to confirm that when men and women are exposed to the same institutional settings there is a small, but observable, difference in how they lead.

### Leadership development for women in STEMM

Through examining leadership traits using the FRLM, researchers have identified that gender differences in leadership styles are observable when context is accounted for and that leadership effectiveness is impacted by followers’ understandings and expectations of gendered leadership styles. Therefore, a possible reason why there is a lower representation of women in leadership positions is that ‘female’ leadership styles are perceived to be less effective compared to ‘male’ leadership styles. To address the gender imbalance in the occupation of leadership positions, a large industry has developed to provide leadership training and development initiatives. Drawing on the observation that there is a difference between men and women in terms of how they lead, and also the effectiveness of their leadership, such initiatives typically implicitly problematise ‘female’ leadership [27–29].

Three common approaches to addressing gender equity have been adopted in leadership training programs–assimilation, accommodation and celebration [30]. Each of these approaches imply that it is women who ‘just don’t fit in’ [30]. Assimilatory approaches encourage and train women to adapt to organisational norms by adopting stereotypically masculine attributes, such as assertiveness and decisiveness [31]. Accommodation approaches typically argue that organisational change is required to address the specific needs of women, such as the provision of maternity leave and formal mentoring programs to compensate for women’s exclusion from informal networks. However, rather than changing organisational norms by providing parental leave and developing inclusive networking practices, women are framed as inherently ‘other’ to these norms [30]. Although celebratory approaches to gender equity have been used to frame women’s ostensibly different attributes as advantageous, these celebratory approaches highlight difference. For example, celebratory approaches position the leadership trait of ‘collaboration’ as a ‘feminine’ trait [4, 17, 18]. The framing of women as ‘other’ shared by all three of these approaches leaves intact existing organisational cultures, in which the structures, processes, and practices reproduce gendered privilege/disadvantage [2, 32]. They also overlook the critical finding that gender differences in leadership and leadership effectiveness are embedded in the organisational and societal context in which it is performed [12, 13, 16].

In examining the factors that determine the effectiveness of leadership development programs, researchers suggest that developing participants’ identities as leaders is a critical element [31–35]. However, internalising an identity as a leader can be difficult for women due to a ‘think manager, think male’ mindset in which leadership is equated with purportedly male attributes such as decisiveness, assertiveness, and independence [2, 36]. As Ely *et al*. observe, ‘what appears assertive, self-confident and entrepreneurial in a man often looks abrasive, arrogant or self-promoting in a woman’ [31]. To facilitate women’s leadership identities, women-only leadership development programs which recognise and address the subtle and pervasive effects of gender bias are required [34, 37, 38]. Harris and Leberman also argue that women-only leadership programs are ‘essential for women to develop a stronger sense of self and stronger relationships to other women’ [37]. To our knowledge, there is minimal research on leadership development programs that focus on the needs of women leaders and virtually no research on emerging women leaders [37].

A review of work on leadership in STEMM reveals that much of the scholarship has focused on describing the nature and extent of gender underrepresentation in leadership. Studies that do examine leadership development for women in STEMM fields are limited to considering ‘pipeline initiatives’ and ‘climate initiatives’ [17]. Pipeline initiatives focus on increasing the leadership capabilities and identities of individual women (e.g. workshops, mentoring, small funding initiatives, networking opportunities). Climate initiatives focus on organisational or structural changes in relation to equity, diversity, and inclusion [27, 39]. For instance, O’Bannon *et al*. studied the effects of a ‘pipeline initiative’–a small leadership programme based in the US Midwest that aimed to help individual women in STEMM fields move into formally recognised academic leadership roles and to travel more effectively through the ‘pipeline’ [17]. In contrast, ‘climate initiatives’ target the problematic masculine culture of STEMM and the associated treatment of women in the academic workforce [39, 40].

While significant evidence describes the organisational factors underpinning the underrepresentation of women in STEMM leadership, it remains that very little is known about how women perform leadership in STEMM fields. Scholars are yet to explore how women in STEMM define leadership and how they perform leadership. Given that gendered leadership styles are contextually dependent, we argue that to address the barriers women face in occupying leadership roles in STEMM, it is first critical to understand how women in STEMM perform and define leadership.

## Methods

This study focused on a group of women in STEMM fields who were seeking to enhance their leadership capacities. The women were participants in a 22-day residential leadership and strategy program for women in STEMM fields administered by an Australian leadership consultancy. The program consisted of eight days of education on best leadership practice, six days of education on leadership challenges in STEMM fields and related issues on climate change, and eight days focussed on the articulation, design, measurement, and execution of strategy. Participants undertook various diagnostic psychometric tests including the Mayer-Salovo-Caruso Emotional Intelligence Test (MSCEIT) and the Life Styles Inventory (LSI) before participating in the program. This program was chosen for this research because it is the only Australian leadership program focused on women in STEMM.

Data for this article were drawn from a pre-program^1^ online survey of registered program participants. The survey was conducted as part of a larger, ongoing mixed methods research project involving pre/post program surveys, qualitative interviews, and video diaries with participants. This article reports only on the pre-program survey. Key research questions in this aspect of the study include:

1. How do women in STEMM define leadership?
2. How do women in STEMM describe their own leadership style?

At the time the study was initiated in 2016, the leadership program had 78 registered participants representing ten nationalities. Despite the apparent diversity of the cohort, all participants were working in industrialised nations and all participants spoke English fluently. All 78 participants were invited to complete the survey. The survey was designed and managed independently from the leadership consultancy and participation was voluntary. The survey was piloted and validated with a group of seven women involved in STEMM leadership prior to distribution [41]. Critically, the survey was distributed prior to participants engaging in leadership or strategy education, diagnostics, or coaching to ensure participants’ reflections on leadership were captured in their own words. The survey contained 17 questions, was hosted on the Qualtrics platform, and distributed by direct email sent on 16 March 2016. An information sheet that provided detail on the background, rationale and anticipated outcomes of the project was included as an attachment to the email. A follow-up email was sent two weeks later and a reminder notice was posted to the private Facebook page for program participants at the same time. The survey was open for one month and closed on 16 April 2016.

Participation in the survey was voluntary. To ensure participants could provide informed consent prior to participating in the study, an electronic consent form was positioned at the start of the questionnaires. A skip logic was used to ensure that any participant who did not provide informed consent could not complete the questionnaire. The study was approved by the University of Tasmania’s Social Sciences Human Research Ethics Committee (Approval: H0015600) as complying with the National Health and Medical Research Council’s (NHMRC) National Statement on Ethical Conduct in Human Research (2007).

Sixty-one of the 78 registered participants completed the survey, a response rate of 78 per cent (no participants dropped out after starting the survey). The survey consisted of both closed and open-ended questions. This format allowed participants to provide unrestricted comments rather than selecting from solely pre-determined choices. Closed questions were used to gather socio-demographic data. Open-ended questions were used to gather data on participants’ perceptions about leadership. Of interest to this article are participants’ responses to the open-ended questions: ‘How do you personally define leadership?’, and ‘What is your leadership style?’

Basic demographic and background information for the sample is provided in Table 2. As shown in Table 2, white middle class women are disproportionately represented in our sample, reflecting white women’s privileged access to leadership positions relative to non-white women [42]. This research limitation supports Lumby and Morrison’s observation that samples of existing leaders are problematic because they are necessarily exclusive and therefore biased [43].

**Table 2:** Participant demographic information.

*Category*	*Number of People*
**Age**	
20–29 years	10
30–39 years	22
40–49 years	14
50–59 years	14
60–69 years	1
**Highest educational level obtained**	
Masters by coursework	5
Masters by research	4
Graduate Certificate	2
Bachelor’s Degree without Honours	10
Bachelor’s Degree with Honours	7
Doctorate by coursework and research	1
Doctorate of Philosophy	32
**Relationship status**	
Married/in a relationship	44
Single	16
Prefer not to say	1
**Racial/ethnic background**	
White	59
White/Asian	2

### Analysis

To identify how the cohort of women in STEMM define and perform leadership, we used the FRLM to determine how the leadership descriptions provided by the women in the sample aligned with the nine factors. Content analysis was used to determine which, if any, of the nine leadership factors women in STEMM identified in relation to their understanding of leadership and their own leadership styles. Qualitative content analysis is a systematic and objective means of making replicable and valid inferences from textual data to their context with the purpose of providing new insight into a phenomenon [44]. The sampling unit was the survey responses and the content unit was leadership. The analysis focussed on manifest and latent content. Manifest content refers to the visible content in the texts and latent content refers to the underlying/hidden meaning of the texts. The combination of manifest and latent content analysis leads to more insightful findings [45].

The content analysis was undertaken in a structured three step process of immersion, reduction, and interpretation [46]. During immersion, the research team engaged with the data before organising it. Survey responses were read repeatedly by all researchers for an overall assessment. Notes were written as we engaged with the data to form a record of initial analytic processes and to aid in the development of a coding scheme. The reduction phase allowed for the development of a systematic approach to the data and the creation of codes. The goal of this phase was to reduce the amount of raw data to that which was relevant to answering the research questions. Codes provided the classification system for the analysis and rigorous review of the data.

Codes were developed using a combination of inductive and deductive approaches, with the deductive codes informed by the FRLM. The descriptions of the nine factors/codes of the FRLM used for the analysis are provided in Table 1. In this stage, multiple coders (members of the research team) highlighted exact words and phrases from the texts that captured key FRLM concepts and overall trends/themes related to leadership. Codes were then sorted into categories based on their relationships and linkages. Categories were used to organise the codes into meaningful groups.

The final stage of analysis involved using the codes to organise the data to enable the researchers to examine patterns that reveal how women in STEMM fields define and perform leadership. Interpretative data summaries were written in relation to data codes and a matrix with analytically meaningful categories was created.

Given the international background of the participants, the analysis was undertaken manually so the authors could account for the use of colloquialisms or contractions to describe particular leadership styles.

We used Pearson’s chi-square test to test if there was any association between FRLM factors and how participants defined leadership and their own leadership styles. Specifically, the data were reviewed to identify if participants clustered factors of the FRLM in their response to the questions ‘How do you personally define leadership?’, and ‘What is your leadership style?

## Results and discussion

### How women in STEMM define leadership

We first assessed participants’ responses to the question about how they personally defined leadership to see how they related to the nine FRLM factors. Some women described multiple factors and some only one. The most frequently described factor was idealised influence as a behaviour, a transformational leadership factor, with 30 participants including it in their definition. This was closely followed by inspirational motivation and idealised influence as an attribute, which was identified by 29 and 27 participants, respectively. In order of frequency of mention, the remaining factors described by the participants were individualised consideration (n = 17), contingent reward (n = 13), active management by exception (n = 7), and intellectual stimulation (n = 4). No participants included passive management by exception or laissez-faire factors in their definitions.

As noted, participants’ definitions of leadership typically included more than one of the nine factors. 13 of the 30 participants who identify idealised influence as a behaviour as an important component of leadership paired this with idealised influence as an attribute. Twelve women paired idealised influence as a behaviour with inspirational motivation. Six participants identify all three factors–idealised influence as an attribute and behaviour and inspirational motivation–in their definitions.

The second most frequently identified leadership factor is inspirational motivation, with 29 participants including this factor within their definition of leadership. Of this group of 29, 12 women also identify idealised influence as an attribute, 12 identify idealised influence as a behaviour, and six identify individualised consideration. As noted earlier, only six participants identify all three of these factors. For those that identify inspirational motivation, there were ten different combinations of factors identified, with two participants identifying five factors in their leadership definitions. While inspirational motivation was most frequently combined with other factors of transformational leadership, five participants who identify inspirational motivation also identify factors associated with transactional leadership.

Intellectual stimulation is the least identified leadership factor (n = 4). Of this group, two participants who include intellectual stimulation in their definitions also include idealised influence as a behaviour and individualised consideration. One participant cites only intellectual stimulation and contingent reward in her definition. The fourth participant includes intellectual stimulation as well as inspirational motivation, idealised influence by behaviour, individualised consideration, contingent reward, and active management by exception.

Table 3 shows the number of times two factors are identified together within participants’ leadership definitions. The Pearson’s chi square test reveals a significant relationship between how the participants regarded contingent reward and idealised influence as an attribute. Only one participant identifies both contingent reward and idealised influence as an attribute, while an equal number of women identify idealised influence as an attribute in their leadership definitions as those who do not. A significant relationship is observed for contingent reward and active management by exception. While most participants identify neither contingent reward nor active management by exception, participants are more likely to identify these factors together than just active management by exception alone. Nevertheless, despite the identification of some patterns in how participants group factors, the results do not reveal strong and uniform relationships among the factors identified by women in STEMM as important to leadership.

**Table 3:** Pairs of factors identified by participants when defining leadership.

	Number of participants who identified pairs of factors, Pearson’s chi square value and p value.						
	Inspirational motivation	Idealised influence as an attribute	Idealised influence as a behavior	Intellectual stimulation	Individualised consideration	Contingent reward	Active management by exception
Inspirational motivation	-	12	12	1	6	4	3
		x = 0.075	x = 0.041	x = 0.753	x = 0.279	x = 2.198	x = 0.030
		p = 0.784	p = 0.839	p = 0.385	p = 0.597	p = 0.138	p = 0.864
Idealised influence as an attribute	12	-	13	0	4	1	0
	x = 0.075		x = 1.028	x = 2.973	x = 1.686	x = 8.603	x = 5.491
	p = 0.784		p = 0.311	p = 0.231	p = 0.194	p = 0.003	p = 0.019
Idealised influence as a behavior	12	13	-	3	11	2	2
	x = 0.041	x = 1.028		x = 1.639	x = 6.814	x = 6.618	x = 0.789
	p = 0.839	p = 0.311		p = 0.200	p = 0.009	p = 0.010	p = 0.374
Intellectual stimulation	1	0	3	-	3	1	1
	x = 0.753	x = 2.973	x = 1.639		x = 5.866	x = 0.010	x = 0.771
	p = 0.385	p = 0.085	p = 0.200		p = 0.015	p = 0.920	p = 0.380
Individualised consideration	6	4	11	3	-	2	1
	x = 0.279	x = 1.686	x = 6.814	x = 5.866		x = 1.040	x = 0.453
	p = 0.597	p = 0.194	p = 0.009	p = 0.015		p = 0.308	p = 0.501
Contingent reward	4	1	2	1	2	-	5
	x = 2.198	x = 8.603	x = 6.618	x = 0.10	x = 1.040		x = 10.509
	p = 0.138	p = 0.003	p = 0.010	p = 0.920	p = 0.308		p = 0.001
Active management by exception	3	0	2	1	1	5	-
	x = 0.030	x = 5.491	x = 0.789	x = 0.771	x = 0.453	x = 10.509	
	p = 0.864	p = 0.019	p = 0.374	p = 0.380	p = 0.501	p = 0.001	

The cluster analysis did not reveal any statistically reliable clusters. This portion of the analysis reveals that while participants typically refer to transformational leadership factors in their leadership definitions, it is not possible to identify one or two definitions of leadership that are ‘typical’ to this group of women.

### How women in STEMM describe their own leadership style

Participants in the study were asked to describe their own leadership style. This question was asked immediately after participants were asked to define leadership. We assessed participant responses to see how their descriptions of their own leadership styles related to the nine FRLM factors. As with participants’ definitions of leadership, their leadership style descriptions often included multiple factors.

The most frequently described factors are idealised influence as a behaviour and individualised consideration (n = 21). While idealised influence as a behaviour is also the most frequently described factor in participants’ definitions of leadership, individualised consideration is only the fourth most frequently identified factor. This finding supports Antonakis *et al*.’s conclusion that women leaders tend to exhibit individualised consideration [24]. Table 4 shows the number of participants who identified each factor in their definitions of leadership and their own leadership style. There appears to be a difference in how women define leadership and how they describe their own leadership style.

**Table 4:** Number of persons referring to each leadership factor in their definition of leadership and description of own leadership style.

Leadership Type	Leadership Factor	Number of participants who identified the leadership factor	
		Definition of leadership	Description of own leadership style
Transformational	Inspirational motivation	29	4
	Idealised influence as an attribute	27	18
	Idealised influence as a behavior	30	21
	Intellectual stimulation	4	2
	Individualised consideration	17	21
Transactional	Contingent reward	13	10
	Active management by exception	7	10
	Passive management by exception	0	0
Laissez-faire	No leadership	0	0

Note: Seven participants who provided a description of idealized leadership were not able, or were unwilling, to describe their own leadership.

Table 5 shows the results of Pearson’s chi-square test for each pair of factors in relation to leadership styles. The results are like the factor parings in leadership definitions (Table 4). The test reveals that there is a significant relationship between how participants regard contingent reward and idealised influence as an attribute. A significant relationship also is evident in relation to contingent reward and active management by exception. Overall, the results do not show significant trends in how participants pair leadership factors within their descriptions of their leadership style.

**Table 5:** Pairs of factors identified by participants when describing their own leadership style.

	Number of participants, Pearson’s chi square value and p value						
	Inspirational motivation	Idealised influence as an attribute	Idealised influence as a behavior	Intellectual stimulation	Individualised consideration	Contingent reward	Active management by exception
Inspirational motivation	-	2	12	0	0	0	0
		x = 0.864	x = 0.168	x = 0.145	x = 2.247	x = 0.839	x = 0.741
		p = 0.353	p = 0.681	p = 0.703	p = 0.134	p = 0.360	p = 0.389
Idealised influence as an attribute	2	-	3	0	3	0	2
	x = 0.864		x = 3.568	x = 0.866	x = 3.568	x = 5.007	x = 0.269
	p = 0.353		p = 0.059	p = 0.352	p = 0.059	p = 0.025	p = 0.604
Idealised influence as a behavior	1	3	-	1	7	1	1
	x = 0.168	x = 3.568		x = 0.222	x = 0.017	x = 3.161	x = 2.542
	p = 0.681	p = 0.059		p = 0.637	p = 0.896	p = 0.075	p = 0.111
Intellectual stimulation	0	0	1	-	1	0	0
	x = 0.145	x = 0.866	x = 0.222		x = 0.222	x = 0.405	x = 0.358
	p = 0.703	p = 0.352	p = 0.637		p = 0.637	p = 0.524	p = 0.550
Individualised consideration	0	3	7	1	-	5	4
	x = 2.247	x = 3.568	x = 0.017	x = 0.222		x = 1.285	x = 0.469
	p = 0.134	p = 0.059	p = 0.896	p = 0.637		p = 0.257	p = 0.493
Contingent reward	0	0	1	0	5	-	4
	x = 0.839	x = 5.007	x = 3.161	x = 0.405	x = 1.285		x = 6.061
	p = 0.360	p = 0.025	p = 0.075	p = 0.524	p = 0.257		p = 0.014
Active management by exception	0	2	1	0	4	4	-
	x = 0.741	x = 0.269	x = 2.542	x = 0.358	x = 0.469	x = 6.061	
	p = 0.389	p = 0.604	p = 0.111	p = 0.550	p = 0.493	p = 0.014	

As with how women described leadership, when reviewing their responses to the question ‘What is your leadership style?’, no reliable clusters are formed. While participants’ descriptions of their leadership style more frequently incorporate transformational leadership factors, it is not possible to identify one or two descriptions of leadership that are ‘typical’ to this group.

### Exploring differences between how women in STEMM define leadership and how they describe their own leadership style

As noted in the previous section, there is a difference in the factors participants identify in their leadership definitions compared to those factors identified in their personal leadership style descriptions. For example:

I see leadership to be the need to inspire and the responsibility to share knowledge. Wherever I go now I see examples of such leadership, even if this is not how it is defined or described, it is the type of leadership that is most respected.

In this definition, the participant emphasises inspirational motivation and idealised influence by behaviour. However, in her definition of her own leadership style, she emphasises individual consideration and intellectual stimulation:

My leadership style is related to what I think a good leader be and do [sic]: To be a good listener; share knowledge; to obtain new learning skills; to be respectful; to take and deliver constructive criticism always in a positive way; to easily understand the contextual situation you are in.

Another participant describes leadership thus:

To be a good leader, you have to be a good listener and to believe in what you are doing so you can enthuse people and take them on the journey with you. No one person has all the skills and experience required to achieve all of the goals of an organisation or team. A good leader has to gather a group of people with diverse skills and experience, encourage, support and listen to everything each person has to offer. Then the leader has to pull together the contributions that each person makes and generate a synthesis of all those efforts to progress the common aim. So a leader has to have the judgement to choose a good team, inspire each member of the team, be supportive so they can do their jobs, have an open mind to listen to what the team contributes, then the ability to pull all those contributions together to achieve the goals of the organisation.

In this extract, intellectual stimulation, individualised consideration, and idealised influence by behaviour are emphasised as important for leadership. Yet in describing her own leadership style, the participant emphasises active management by exception:

I am the focal point of a collaborative team, pulling together the threads to generate a cohesive output. I ensure all voices are heard, however where necessary I will make a call as to what we will do. I encourage subgroups of the team to work together.

While all participants in the survey could define leadership, seven participants were not able to describe their leadership style. Some noted that perhaps after participating in the leadership program they would be in a better position to describe their leadership style. For example:

Leadership is about entrenching ethics and morals in the people that are working for you. About encouraging and bringing out of people their strengths as well as identifying and developing weaknesses. It is about bringing together a group of people and optimising their output for a collective agenda.

However, when describing her own leadership style, she noted: “I'm not really sure”.

Although the definitions of leadership discussed above appear to be gender-neutral, it is possible that the women’s assessments of their own leadership styles are influenced by the profile of those holding leadership positions in their organisational contexts. For instance, it is well known that women in STEMM fields have far fewer role models and less social support when it comes to leadership. Women in traditionally male-dominated fields generally have a much smaller pool of high-status women to network with and they also have less contact with influential men in their fields [31]. Indeed, more than 50 per cent of the participants commented that they applied for the leadership program for the opportunity to network with other women leaders:

The prospect of developing my leadership and strategic planning skills in a focussed and supportive environment with passionate and like-minded women …was an opportunity that I could not overlook.

Even though the program includes women who are leaders or emerging leaders in STEMM, and indeed, several of the participants occupy very senior leadership roles (e.g. head of research group; chair of faculty; director of company), most survey participants do not see their leadership styles as being that of the idealised leader. In this respect, most participants are typical of high performing women who tend to see themselves as lacking the ‘raw materials’ for leadership [47]. However, it is notable that the participants identify individual reasons for applying to the program and not structural/organisational ones. In this sense, women still tend to look to improve their perceived personal failings without recognising the gendered organisational climate in which they were working.

## Conclusion

While our survey respondents are similar in that they all are existing or emerging leaders in STEMM and are from similar race/class/cultural backgrounds, there is less agreement in terms of how they define leadership or describe their own leadership style. Across the group there is a tendency for participants to use transformational leadership factors to define leadership and their own leadership styles. However, most emphasise only 2 or 3 of the factors and while some factors are more frequently noted by participants, there is no observable pattern to how participants cluster these factors. Over 25 per cent of participants also associate leadership with transactional factors. None of the participants define leadership with reference to all the transformational or transactional leadership factors. Likewise, none of the participants describe their leadership style using all the transformational and transactional leadership factors. There is also little consistency in terms of how participants group leadership factors with reference to their definitions of leadership and their own leadership styles. Furthermore, there is no discernible pattern to how participants define leadership as compared to how they describe their own leadership style. These findings challenge popular conceptions of a distinct female style of leadership. We anticipate that future research (e.g. analysis of a post-program survey) may provide more clarity on some of these points including how organisational context influences leadership styles.

The qualitative analysis of participants’ comments reveal how the participants understood relational/interpersonal aspects of leadership (e.g. caring for others, building a team, active listening) that are often expected of women as leaders [48]. It is possible that valuing particular leadership factors over others is reflective of participants’ knowledge that to be ‘legitimate’ leaders in STEMM they must avoid performing stereotypical masculine leadership styles [49]. It might also be related to cultural expectations that women will enact a stereotypical female leadership style which is more participatory/collaborative [50, 51].

Previous research has revealed that the effectiveness of leadership style, irrespective of gender, is dependent on the context in which it is being exercised. While it is widely recognised that there is an underrepresentation of women in leadership in STEMM fields, very little is known about how women in STEMM fields perform leadership and, in turn, if this relates to the gender imbalance. Drawing on studies that examine gender difference, legitimacy, and effectiveness in organisations, behaviours that are effective for men in STEMM contexts may not be effective for women. This study reveals that women in STEMM value and perform selected transformational leadership factors more so than transactional leadership factors. However, tellingly, while the participants in the study identify inspirational motivation (a transformational leadership factor) as an important leadership component, only four women indicate that they practice this traditionally ‘masculine’ trait. In contrast, many more women in the study are engaged in idealized influence (a transformational leadership factor) as a behaviour such that they identify themselves as members of collaborative teams, a stereotypically female leadership behaviour. A critical point for women, then, is that organisational contexts and/or leadership expectations are gendered and recognising gendered variations in organisational contexts may be key to enabling women to be effective leaders [52]. Further research is needed about how women lead, why they lead in particular ways and the effectiveness of different leadership styles in STEMM contexts.

### Notes

1. The post-program survey will be conducted one-year post-program and will enquire about participants’ perceived usefulness of the program, changes in their understandings of leadership, and contextual factors.

## Supporting information

S1 File Questionnaire. (PDF)
