# Supplementary material for: What style of leadership do women in STEMM fields perform? Findings from an international survey
Source: PLoS One. 2017 Oct 5;12(10):e0185727. doi: 10.1371/journal.pone.0185727 (PMC5628874; doi:10.1371/journal.pone.0185727)
Supplement: S1 File — (PDF) [file pone.0185727.s001.pdf]

## Consent to participate

**1. I have read and understand the Information Sheet for this project.**

**2. The nature and possible effects of the study have been explained to me.**

**3. I understand that the study involves completing an online survey. I understand that all data collected will take place with a focus on how Homeward Bound participants define leadership and how they invest in leadership training.**

**4. I understand that all research data will be securely stored on the University of Tasmania premises for five years, and then will be securely destroyed.**

**5. Any questions that I have asked have been answered to my satisfaction.**

**6. I agree that research data gathered from me for the study (i.e. data obtained from the survey) may be published provided that I cannot be individually identified as a participant.**

**7. I understand that I will not be asked to identify myself on the questionnaire form and that any information I supply to the researcher will be used only for the purposes of the research.**

**8. I agree to participate in this investigation and understand that I may withdraw at any time without any effect, and if so wish, may request that any data I have supplied to date be withdrawn from the research.**

**I have read the Terms and Conditions above and consent to participate in this project by completing this survey.**

- ◯ Yes
- ◯ No

## Homeward Bound and leadership

**In one sentence, explain why you applied for homeward bound?**

**How do you personally define leadership? (please tell us in under 200 words)**

**What is your leadership style? (under 50 words)**

## Factors influencing your decision to apply

**Thinking back to when you first heard about Homeward Bound, how important were each of the following factors in your decision to apply?**

| | Very important | Moderately important | Not important |
|---|---|---|---|
| 1. To develop my leadership skills | ◯ | ◯ | ◯ |
| 2. To develop my leadership skills for the purpose of influencing policy for a more sustainable world | ◯ | ◯ | ◯ |
| 3. To develop skills to progress my career | ◯ | ◯ | ◯ |
| 4. To improve my strategy and execution skills | ◯ | ◯ | ◯ |
| 5. To travel to Antarctica | ◯ | ◯ | ◯ |
| 6. To learn about polar science | ◯ | ◯ | ◯ |
| 7. To be involved in a documentary | ◯ | ◯ | ◯ |
| 8. Exposure to the global faculty | ◯ | ◯ | ◯ |
| 9. Forming networks with other participants | ◯ | ◯ | ◯ |
| 10. To support other women pursuing leadership in science | ◯ | ◯ | ◯ |

## Factors negatively influencing decision to apply

### Did any of the following factors cause you concern when you applied for Homeward Bound?

| | Very concerning | Moderately concerning | Not of concern |
|---|---|---|---|
| Making a Youtube video for the application | ○ | ○ | ○ |
| Committing to raise the funds for your participation | ○ | ○ | ○ |
| The timing of the journey with the holiday season | ○ | ○ | ○ |
| Possibility of seasickness | ○ | ○ | ○ |
| Coping with the weather in Antarctica | ○ | ○ | ○ |
| Sharing accommodation/ close living quarters on the ship | ○ | ○ | ○ |
| Securing/ taking time of work or annual leave | ○ | ○ | ○ |
| Pregnancy plans | ○ | ○ | ○ |
| Family responsibilities | ○ | ○ | ○ |
| Lack of support from family / partner | ○ | ○ | ○ |
| Lack of support from your employer | ○ | ○ | ○ |
| Being filmed for the documentary | ○ | ○ | ○ |

### If you wish, please elaborate on any of the concerns you had when you applied. This may include factors not listed in the previous question.

## About your funding

### Have you met your funding goal

○ Yes

○ No

### Considering the full program and trip costs (participation fee, flights, accommodation, insurance, clothing) please indicate the percentage of funding you have (or are hoping to) secure from each source. The total must equal 100%

| | |
|---|---|
| Self-funding | 0 |
| Employer sponsorship | 0 |
| Accumulated funds in academic cost centre (from awards, grants etc.) | 0 |
| Corporate sponsorship | 0 |
| Fundraising (including crowd-funding) | 0 |
| External funding/ grants | 0 |
| Other | 0 |
| **Total** | 0 |

### If you wish, this space is to tell us more about your experience in securing funding for this trip. You can write as little or as much as you wish in this space.

## Demographics

### Please indicate your age

- 20-29 years
- 30-39 years
- 40-49 years
- 50-59 years
- 60-69 years
- 70+

### What is your highest attained educational level

- Highschool
- Highschool/ currently completing University Bachelors Degree
- Bachelors Degree without honours
- Bachelors Degree with honours
- Graduate Certificate
- Masters by coursework
- Masters by research
- Doctorate by coursework and research
- Doctorate of Philosophy

### What is your nationality?

### What is your current country of residence?

### How would you describe your ethnicity/ cultural background?

### Please indicate the sector of your main employment

- Private
- Government
- University

### What is your annual salary (including bonuses and commissions) in U.S. dollars?

- $0 - $25,000
- $25,001 - $50,000
- $50,001 - $75,000
- $75,001 - $100,000
- $100,001 - $125,000
- $125,001 - $150,000
- $150,001 - $175,000
- $175,001 - $200,000
- $200,001+

### Please indicate your relationship status

- ◯ Married in a registered marriage
- ◯ Married in a defacto marriage
- ◯ Not married

**How would you describe your sexuality?**

**Please briefly tell us about any caring responsibilities you have. This may include for children or adults.**

**Do you have any additional comments?**
